# High-expression of BCL10 inhibits cell-mediated immunity within the tumor immune microenvironment

**DOI:** 10.3389/fimmu.2025.1616321

**Published:** 2025-06-05

**Authors:** Jinyi Gu, Changshun Chen, Yuanjing Chen, Wei Lv, Fei Li, Puyuan Zhu, Pu He, Yunjie Du, Huiling Liu, Bingdong Zhu

**Affiliations:** ^1^ Gansu Provincial Key Laboratory of Evidence Based Medicine and Clinical Translation, Institute of Pathogen Biology, School of Basic Medical Sciences, Lanzhou University, Lanzhou, China; ^2^ Clinical Laboratory, Affiliated Hospital of Yunnan University, Kunming, China; ^3^ Department of Orthopedics, Lanzhou University Second Hospital, Lanzhou, China; ^4^ Department of Orthopedics and Trauma Surgery, Affiliated Hospital of Yunnan University, Kunming, China; ^5^ The First Clinical Medical College of Gansu University of Chinese Medicine, Lanzhou, China; ^6^ The First School of Clinical Medicine, Lanzhou University, Lanzhou, China; ^7^ Department of Gynecology and Obstetrics, Gansu Provincial Hospital, Lanzhou, China

**Keywords:** B-cell lymphoma 10 (BCL10), tumor immune microenvironment (TIME), CD8+T cells, cervical squamous cell carcinoma(CESC), NF-κB signaling pathway, PD-1

## Abstract

**Background:**

B-cell lymphoma 10 (BCL10) is a key signaling molecule that plays a pivotal role in activating the NF-κB signaling pathway. However, it is unclear whether prolonged activation of BCL10 within tumors lead to T cell exhaustion and suppress immune responses against cancer.

**Methods:**

In this study, through multi-layered bioinformatics analysis and experimental verification, the role of BCL10 on immune cells in the tumor immune microenvironment was systematically investigated. The correlation between BCL10 expression and infiltrated immune cells was analyzed by immune algorithms such as xCELL, CIBERSORT, QUANTISEQ and MCPcounter using TCGA and GTEx databases. Furthermore, single-cell RNA sequencing data of the cervical squamous cell carcinoma(CESC)microenvironment were analyzed using the GEO database. Moreover, an implanted CESC mice model was established, in which the expression and correlation of BCL10, NF-κB, and PD-1 in CD8+ T cells, as well as the proliferation and apoptosis of CD8+ T cells, were analyzed.

**Results:**

In silico analysis revealed that the upregulation of BCL10 in tumor immune microenvironment (TIME) was correlation with decreased infiltration of CD8+T cells, CD4+Th1 cells and NK T cells, and increased infiltration of Treg cells and CD4+Th2 cells in most tumors including CESC. In implanted CESC mice model, BCL10 expression was found upregulated in CD8+T cells, consequently activating the NF-κB signaling, and leading to upregulation of PD-1 expression, inhibiting proliferation of CD8+T cells, and promoting apoptosis of CD8+T cells.

**Conclusions:**

BCL10 is closely associated with immunosuppression across various tumor types. In CESC, chronic stimulation and over-activation by tumor antigens result in exhaustion of CD8+ T cells through the over-activation of the NF-κB signaling pathway and upregulation of PD-1 expression.

## 1 Background

The tumor immune microenvironment (TIME), being critical in regulating tumor initiation, progression, and therapeutic responses (1, 2), consisting of CD8+ T cells, CD4+ T helper cells, regulatory T cells (Tregs), B cells, natural killer T (NKT) cells, and tumor-associated macrophages (TAMs) (3). Among them, CD8+ T cells and NK cells are critical effector cells in anti-tumor immunity, directly eliminating tumor cells through cytotoxic activity. CD8+ T cells recognize tumor-specific antigens presented on MHC class I molecules *via* T cell receptors (TCRs), triggering the release of cytotoxic molecules (e.g., perforin, granzyme) to induce tumor cell apoptosis (4). Additionally, they produce cytokines such as IFN-γ and TNF-α, which enhance immune responses by upregulating antigen presentation and inhibiting tumor angiogenesis through VEGF downregulation (5, 6). This dual function of direct cytotoxicity and immune modulation establishes CD8+ T cells as central regulators of antitumor immunity. CD4+ Th1 cells further bolster anti-tumor immunity by secreting pro-inflammatory cytokines (e.g., IFN-γ) and promoting the activation and functionality of CD8+ T cells (7). Preclinical evidence also implicates NKT cells in mediating anti-tumor activity (8). Conversely, immunosuppressive cell subsets within the TIME promote tumor aggressiveness. For instance, CD4+ Th2 cells skew the immune response toward tumor promotion by suppressing CD8+ T cell activation and fostering tumor invasion and metastasis, as evidenced in breast and pancreatic cancer models (9).Similarly, Tregs exacerbate immunosuppression *via* inhibiting Th1 differentiation and CD8+ T cell effector functions, thereby dampening the host’s anti-tumor immune responses (10).

Tumor immunity progresses through three stages: Elimination (immune system attacks tumor cells), Equilibrium (tumor and immune system coexist), and Escape (tumor evades immune control) (11). During the escape phase, T cell exhaustion—characterized by persistent PD-1 (programmed death receptor-1) upregulation, loss of effector functions, and impaired proliferation—becomes a critical barrier to antitumor immunity (12–14). Normal tissues have low expression of PD-L1 and are only upregulated briefly in response to inflammation or infection (15). Within the tumor microenvironment, Tumor cells robustly upregulate PD-L1 expression via mechanisms such as genetic mutations, hypoxia, and inflammatory stimuli (e.g., IFN-γ) the PD-1/PD-L1 signaling pathway is exploited to facilitate immune evasion (16, 17). PD-1 signaling not only inhibits T cell activity but also reinforces exhaustion by promoting the transcription of exhaustion-associated genes, such as TOX and Blimp-1 (18). When PD-L1 on tumor cells binds to PD-1 on T cells, this interaction suppresses T cell proliferation, cytokine secretion (e.g., IFN-γ, TNF-α), and cytotoxic activity, ultimately leading to T cell dysfunction or exhaustion (19, 20). For example, after PD-1 binds to PD-L1 on the surface of tumor cells, it inhibits the activation and function of CD8+T cells and promotes tumor immune escape (21). Furthermore, sustained PD-L1 blockade paradoxically drives the recruitment of immunosuppressive cells, such as regulatory T cells (Tregs) and tumor-associated macrophages (TAMs), thereby further suppressing anti-tumor immunity (22).

At the same time, the transcription factor NF-κB plays a dual role in tumor immunity progresses: although it initially promotes anti-tumor T cell response during the elimination phase, long-term tumor antigen stimulation overstimulates NF-κB, driving PD-L1 expression and exacerbating T cell exhaustion (23, 24). Recent studies further reveal that NF-κB signaling in exhausted T cells sustains their survival but compromises their functionality, creating a feedback loop that perpetuates immune evasion (24, 25). Additionally, NF-κB-mediated upregulation of CXCR5 in exhausted CD8+ T cells traps them in tumor-draining lymph nodes, further limiting their antitumor activity (26, 27). BCL10 (B-cell lymphoma 10) is a key signaling molecule critical for immune cell activation (28). As a central component of the CBM complex CARMA1-BCL10-MALT1) and a critical regulator of the NF-κB pathway, it drives NF-κB activation, modulating immune cell function and activity (29, 30). The CBM complex (CARMA1-BCL10-MALT1) bridges antigen receptor signaling to NF-κB activation through three key steps: (1) Antigen stimulation triggers CARD11 phosphorylation and oligomerization; (2) BCL10 forms filaments that recruit MALT1; (3) MALT1 activates IKK to degrade IκBα, enabling NF-κB nuclear translocation and target gene expression (e.g., IL-2, PD-1) (31–36). In the TIME, the activation of NF-κB signaling in both tumor cells and immune cells can upregulate PD-L1 expression, thereby facilitating immune evasion by suppressing T cell activity (37–39). Recent studies have shown that BCL10 is abnormally expressed in various tumors and may influence tumor progression by regulating the immune microenvironment (40–42). As a critical effector downstream of BCL10 activation, NF-κB signaling simultaneously promotes tumor cell survival and proliferation while suppressing antitumor immunity through immune cell dysregulation, thereby enabling tumor immune evasion (43). Excessive NF-κB activation may induce T cell exhaustion, particularly within the TIME (44, 45).

Using bioinformatics analysis, we found that BCL10 is highly expressed in the TIME of most tumors, and its elevated expression correlates with immunosuppression within the TIME. This was validated *via* multi-omics analysis, scRNA-seq analysis, and implanted CESC mice experiment. It indicated that elevated BCL10 is associated with exhaustion of T cell in TIME.

## 2 Materials and methods

### 2.1 The correlation analysis of BCL10 expression and immune score

STAR-counts data and corresponding clinical information for 33 different types of cancers (**Box 1**) were downloaded from the TCGA database (https://portal.gdc.cancer.gov). Data in TPM (transcripts per million) format were extracted, and normalization was performed using the log2(TPM+1) transformation. Only samples containing both RNA-seq data and clinical information were retained for further analysis.

Box 1 **Abbreviations and details of the 33 cancer types used in this study.**

**Table:** 

Abbreviation	Detail
ACC	Adrenocortical Carcinoma
BLCA	Bladder Urothelial Carcinoma
BRCA	Breast Invasive Carcinoma
CESC	Cervical Squamous Cell Carcinoma
CHOL	Cholangiocarcinoma
COAD	Colon Adenocarcinoma
DLBC	Lymphoid Neoplasm Diffuse Large B Cell Lymphoma
ESCA	Esophageal Carcinoma
GBM	Glioblastoma
LGG	Brain Lower Grade Glioma
HNSC	Head And Neck Squamous Cell Carcinoma
KICH	Kidney Chromophobe
KIRC	Kidney Renal Clear Cell Carcinoma
KIRP	Kidney Renal Papillary Cell Carcinoma
LAML	Acute Myeloid Leukemia
LIHC	Liver Hepatocellular Carcinoma
LUAD	Lung Adenocarcinoma
LUSC	Lung Squamous Cell Carcinoma
MESO	Mesothelioma
OV	Ovarian Serous Cystadenocarcinoma
PAAD	Pancreatic Adenocarcinoma
PCPG	Pheochromocytoma And Paraganglioma
PRAD	Prostate Adenocarcinoma
READ	Rectum Adenocarcinoma
SARC	Sarcoma
SKCM	Skin Cutaneous Melanoma
STAD	Stomach Adenocarcinoma
TGCT	Testicular Germ Cell Tumors
THCA	Thyroid Carcinoma
THYM	Thymoma
UCEC	Uterine Corpus Endometrial Carcinoma
UCS	Uterine Carcinosarcoma
UVM	uveal melanoma

To perform an accurate immunological assessment, we used four new immune cell concentration estimation algorithms—xCELL, quanTIseq, MCP-counter, and CIBERSORT—to analyze the correlation between BCL10 expression and immune infiltration across multiple cancers.

Statistical analysis was conducted using R software, version v4.0.3. Results were considered statistically significant when the *p*-value was less than 0.05.

### 2.2 Correlation between BCL10 expression and immunomodulatory genes

Immunedeconv, an R software package integrating the latest algorithms TIMER and xCell, was utilized for robust immunization scoring and evaluation. The computational Spearman correlation analysis heat map was generated to depict the relationship between immune BCL10 gene expression and checkpoint-related genes across multiple cancers. Additionally, gene co-expression analyses were conducted to investigate the interplay between BCL10 expression and immune-related genes in pan-cancer. The uniformly normalized pan-cancer dataset, TCGA TARGET GTEx (PANCAN, N=19131, G=60499), was obtained from the UCSC database (https://xenabrowser.net). Subsequently, the BCL10 gene and expression data of 150 marker genes representing five immune pathways (chemokine (41), receptor (18), MHC (21), immune inhibitor (24) and immunostimulator (46) were extracted from each sample. Normal samples were filtered and log2 transformation was applied to normalize the expression values. Pearson correlation coefficients were computed between BCL10 and the marker genes of the five immune pathways. The resulting heat map displayed different cancer types on the horizontal axis, various immunization scores on the vertical axis, and distinct colors representing correlation coefficients.

Based on the BCL10 expression in CESC cancer, patients were categorized into high-expression and low-expression groups. The influence of BCL10 on the expression of common immune checkpoints (CD274, CTLA4, HAVCR2, LAG3, PDCD1, PDCD1LG2, TREAM and SIGLEC15) was evaluated to measure the potential link between BCL10 expression and response to immunotherapy.

Statistical analysis was conducted using R software, version v4.0.3. Results were considered statistically significant when the p-value was less than 0.05.

### 2.3 Single-cell transcriptome sequencing data analysis

Downloaded from GEO database (https://www.ncbi.nlm.nih.gov/geo) and analysis (GSE168652) single cell sequencing data set. Use the R software MAESTRO and Seurat to process and analyze the single-cell data. Re-cluster the cells using the t-SNE method. Downloaded STAR-counts data and corresponding clinical information for CESC tumors from the TCGA database (https://portal.gdc.cancer.gov). The data in TPM format was then extracted by us, and normalization was performed using the log2(TPM+1) transformation. Samples containing RNAseq data and clinical information were retained for analysis.

The genes included in the corresponding pathways were collected by us, and then they were analyzed using the GSVA package in R software, with the parameter method = ‘ssgsea’ chosen for single-sample gene set enrichment analysis (ssGSEA). Finally, the correlation between gene expression and pathway scores was studied through Spearman correlation analysis.

### 2.4 Association of BCL10 expression with TMB and MSI

The Tumor Mutational Burden (TMB) and Microsatellite Instability (MSI) scores were extracted from the TCGA dataset (https://portal.gdc.cancer.gov). Spearman’s method was utilized to perform a correlation analysis to investigate the association between BCL10 expression and TMB, as well as MSI. The graph depicts the correlation coefficient between BCL10 and TMB/MSI on the horizontal axis, with different types of cancer represented on the vertical axis. The size of the dots in the graph corresponds to the magnitude of the correlation coefficient, while different colors indicate the significance of the p-value, with bluer colors indicating smaller *p*-values.

### 2.5 Immunohistochemistry staining

Immunohistochemistry (IHC) images were obtained from the Human Protein Atlas (HPA) database (http://www.proteinatlas.org) to evaluate variations in BCL10 protein expression in CESC. The images were analyzed for differences in BCL10 protein expression.

### 2.6 Models of implanted and metastatic CESC in mice

A total of 20 female C57BL/6J mice, aged 6–8 weeks, were purchased from Lanzhou University (Gansu, China) and randomly assigned into four groups (n = 5 per group): control group 1, control group 2, CESC group 1, and CESC group 2. To establish implanted CESC and metastatic CESC tumor mouse models, 5 × 10^5 TC-1 tumor cells in 100 μL were injected into the inguinal region of mice in the CESC group 1 and into the abdominal cavity of mice in CESC group 2, respectively. Mice in the control groups were administered an equivalent volume of PBS. All animal experiments were conducted in accordance with the guidelines of the Council on Animal Care and Use, and the protocols were reviewed and approved by the Institutional Animal Care and Use Committee of Lanzhou University.

### 2.7 Mouse CD8+T cells were sorted to analyze BCL10 expression, NF-κB activation, cell proliferation and apoptosis

Lymphocyte single-cell suspensions were prepared at a concentration of 1 × 10^8 cells/ml. To enrich CD8+ T cells, 200 µL of MagniSort™ Enrichment Antibody Cocktail (MagniSort, Thermo Fisher, China) was added to the cell suspensions, mixed thoroughly, and incubated for 10 minutes at room temperature. The cells were then washed, and 200 µL of MagniSort™ Negative Selection Beads (MagniSort, Thermo Fisher, China) was added. After incubation at room temperature for 5 minutes, the cells were washed again. The tube was inserted into a magnet and incubated for an additional 5 minutes at room temperature. The cell suspension that flowed through the magnet into a new test tube contained the isolated CD8+ T cells.

The BeyoClick™ EdU-488 staining assay (Beyotime, Shanghai, China) was used to analyze cell proliferation. Briefly, inoculated cells were labeled with the prepared EdU solution and incubated for 2 hours. The cells were then washed twice with PBS, fixed with 4% paraformaldehyde, and incubated with PBS containing 0.3% Triton X-100 for 15 minutes. Subsequently, the cells were incubated with the prepared Click Reaction Solution for 30 minutes. Finally, cell proliferation was assessed by flow cytometry (NovoCyte Quanteon, Beijing, China), with cells not labeled with EdU serving as a negative control.

After 48 hours of cell transfection in a 6-well plate, the cell culture medium was removed, and the cells were washed once with PBS. Following the protocol of the Annexin V-FITC Cell Apoptosis Detection Kit (Yeasen, Shanghai, China), Annexin V-FITC binding solution was added to the cells, followed by the addition of Annexin V-FITC and propidium iodide staining solution. The mixture was gently mixed and incubated in a light-avoiding environment at room temperature for 15 minutes. Apoptosis was then detected by flow cytometry (NovoCyte Quanteon, Beijing, China).

### 2.8 Western blot

Cells in the logarithmic growth phase were collected and lysed on ice for 30 minutes using RIPA buffer (Solarbio, Beijing, China) containing 1 mM PMSF. The lysate was then centrifuged to obtain the supernatant. The protein concentration in the samples was determined using a BCA protein assay kit (Solarbio, Beijing, China). The protein samples were mixed with loading buffer, heated at 100°C for 10 minutes to denature the proteins, and then loaded onto a 10% SDS-polyacrylamide gel for electrophoresis. The proteins were subsequently transferred onto a polyvinylidene fluoride (PVDF) membrane (Millipore, USA). After a 20-minute incubation in 5% skimmed milk powder TBST buffer (membrane sealing solution), the membrane was incubated sequentially with primary antibodies and HRP-conjugated secondary antibodies. Finally, the target proteins were visualized using the ECL High Sensitivity Chemiluminescent Substrate (Biosharp, Anhui, China).

The antibodies used were as follows: β-actin (1:50,000, 66009-1-Ig), BCL10 (1:3000, 66556-1-Ig), HRP-conjugated anti-mouse IgG (1:5000, SA00001-1), and anti-rabbit IgG (1:5000, SA00001-2) were obtained from Proteintech (Hubei, China). NF-κB p65 (1:1500, T55034) and phospho-NF-κB p65 (Ser536) (1:1000, TP70621) were obtained from Abmart (Shanghai, China).

### 2.9 Flow cytometry analysis for the expression of PD-1 on CD4+ or CD8+ T cells

Lymphocytes were isolated from the spleen or Lymph node of normal and tumor-bearing mice by using Mouse 1× Lymphocyte Separation Medium (Dakewe, Shenzhen, China) and cultured in media containing RPMI-1640, 10% fetal bovine serum (FBS), 100 U/ml Penicillin-Streptomycin Solution. Then the lymphocytes were inoculated in 24 well plates at the number of 5*106 cells/well. Single-cell suspensions were generated in PBS buffer with 1% FBS and 0.1 mM EDTA. Cells were incubated with FITC Anti-Mouse CD4 antibody (Clone RM4-5, Biolegend, USA), PerCP-Cyanine5.5 Anti-Mouse CD8a antibody (Clone 53-6.7, Biolegend, USA) and PE-Hamster Anti-Mouse CD279 antibody (Clone J43, BD Biosciences, USA) for 30 min at 4 °C in the dark, then the cells were washed twice with PBS buffer containing 1% FBS and 0.1 mM EDTA. Detection was performed by flow cytometry (NovoCyte Quanteon, Beijing, China).

### 2.10 Statistical analysis

T-tests were performed to assess the differential expression of BCL10 in cancerous and normal tissues. The experimental data were expressed as the mean ± standard deviation (SD) of 3 independent experiments. All statistical analyses were performed using GraphPad Prism 5.2 software (GraphPad Software, Inc.). The differences between the two groups were analyzed by Student’s t test. A significance threshold of p<0.05 was applied to all statistical analyses.

## 3 Results

### 3.1 Overexpression of BCL10 inhibits immune cell infiltration in TIME

**Figure 1:**
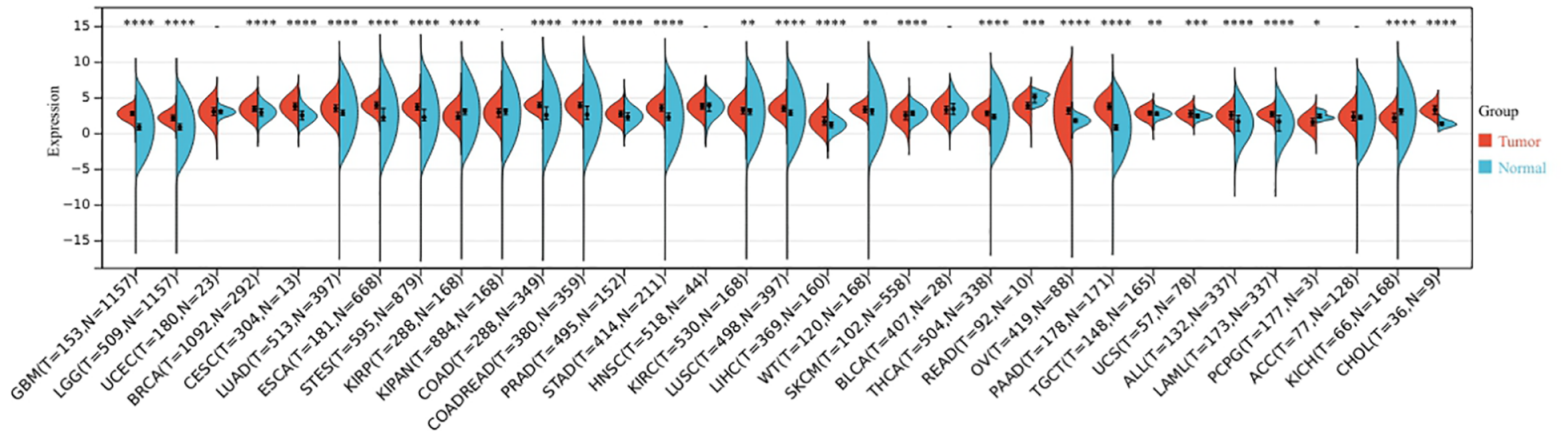
The expression profile of BCL10 in the tumor microenvironment. BCL10 expression was detected in the tumor microenvironment across 33 distinct types of tumors. The x-axis shows the different tumor types, red represents the tumor group, blue represents the normal control group. The y-axis represents the expression of BCL10. *p < 0.05, **p < 0.01, ***p < 0.001, ****p < 0.0001 with asterisks indicating the level of significance.

The expression levels of BCL10 in normal and tumor tissues were compared from the TCGA and GTE databases. The results indicated that BCL10 expression was up-regulated in the majority of human cancers, including ALL, BRCA, CESC, CHOL, COAD, ESCA, GBM, KIRC, LAML, LGG, LIHC, LUAD, LUSC, OV, PAAD, PRAD, STAD, STES, THCA, TGCT, UCS, and WT tumors (**Figure 1**).

**Figure 2:**
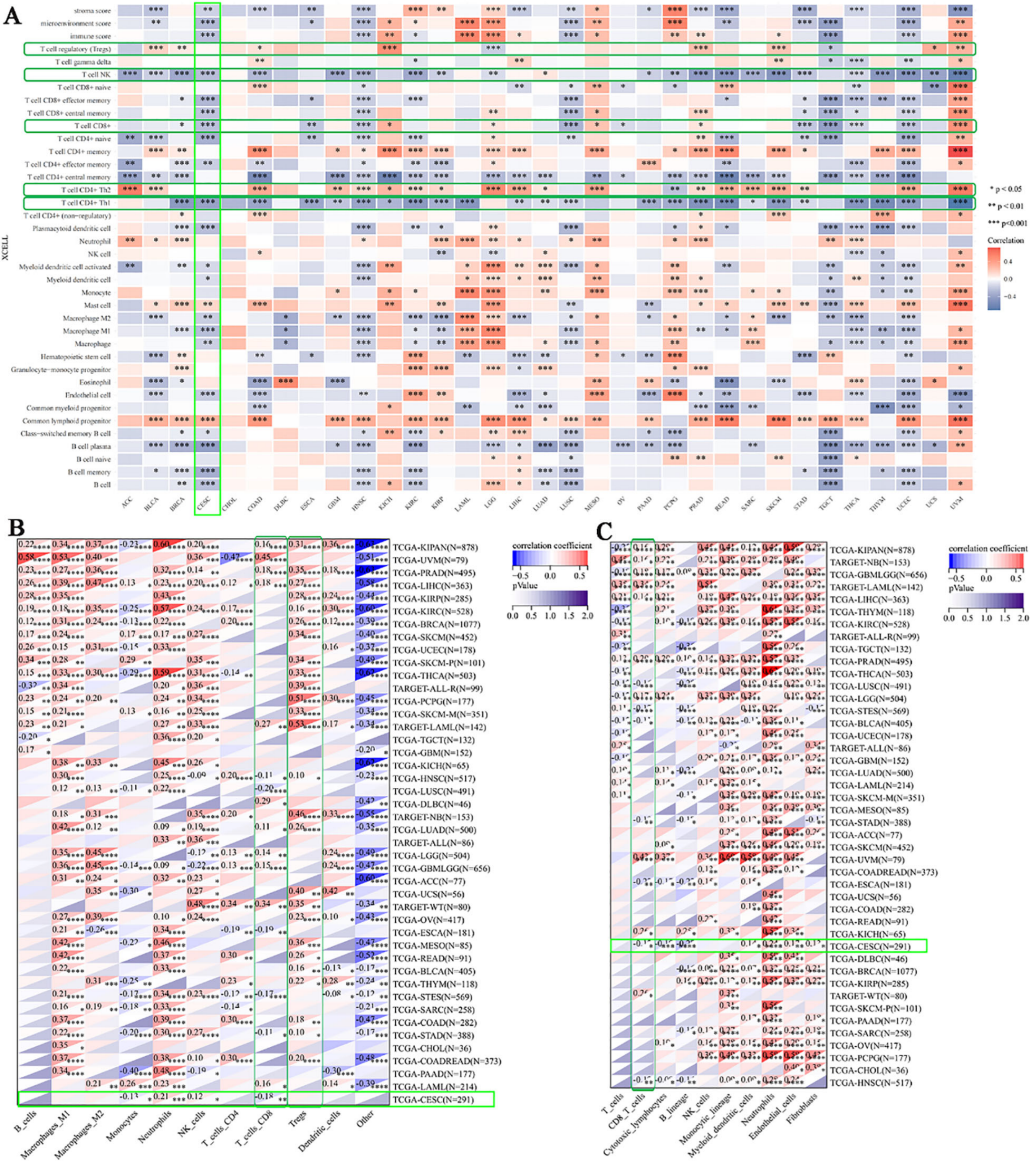
Correlation between BCL10 expression and immune cell infiltration. Heatmap of pertinence between the expression of BCL10 and immune infiltration in 33 types of cancer using xCELL **(A)**, QUANTISEQ **(B)** and MCPcounter **(C)**. The x-axis represents different tumor tissues, and the y-axis represents different immune infiltration scores. Different colors represent the correlation coefficients, with negative values indicating negative correlation and positive values indicating positive correlation. The stronger the correlation, the deeper the color. *p < 0.05, **p < 0.01, ***p < 0.001, ****p < 0.0001 with asterisks indicating the level of significance.

To comprehensively evaluate the correlation between BCL10 expression and the tumor immune microenvironment (TIME) and immune cell infiltration, we quantified tumor-infiltrating immune cells from RNA sequencing data using four algorithms: xCELL, QUANTISEQ, MCPcounter, and CIBERSORT. By integrating the results from these four algorithms, we discovered that BCL10 expression was negatively correlated with tumor-suppressing immune cells, including CD8+ T, CD4+ Th1, and NKT cells, and positively correlated with tumor-promoting immune cells, such as CD4+ Th2 and Treg cells (**Figure 2**, **Supplementary Figure 1**). The xCELL algorithm revealed significant correlations between BCL10 expression and the infiltration of various immune cells across multiple tumors. Specifically, CD8+ T cell infiltration was significantly negatively correlated with BCL10 expression in 10 tumors. CD4+ Th1 cell infiltration was significantly negatively associated with BCL10 expression in 24 tumors. NKT cell infiltration was significantly negatively associated with BCL10 expression in 22 tumors. Additionally, Treg infiltration was significantly positively associated with BCL10 expression in 8 tumors. Furthermore, CD4+ Th2 cell infiltration was significantly positively associated with BCL10 expression in 19 tumors (**Figure 2A**). The QUANTISEQ algorithm showed that the infiltration of CD8+T cells was significantly negative correlation with BCL10 expression in 6 tumors, and the infiltration of Tregs was significantly positively associated with BCL10 expression in 26 tumors (**Figure 2B**). The MCP algorithm showed that the infiltration of CD8+ T cells was significantly negative correlation with BCL10 expression in 7 tumors (**Figure 2C**). Furthermore, the CIBERSOR algorithm showed that the infiltration of CD8+T cells was significantly negative correlation with BCL10 expression in 14 tumors (**Supplementary Figure 1**). Notably, results from four algorithms indicated that BCL10 was significantly negatively correlated with CD8+T cells in CESC. These results suggest that BCL10 may inhibit tumor immunity mainly by inhibiting CD8+T cells in the immune microenvironment of CESC.

### 3.2 Relationship between BCL10 and immune checkpoint, MSI and TMB

**Figure 3:**
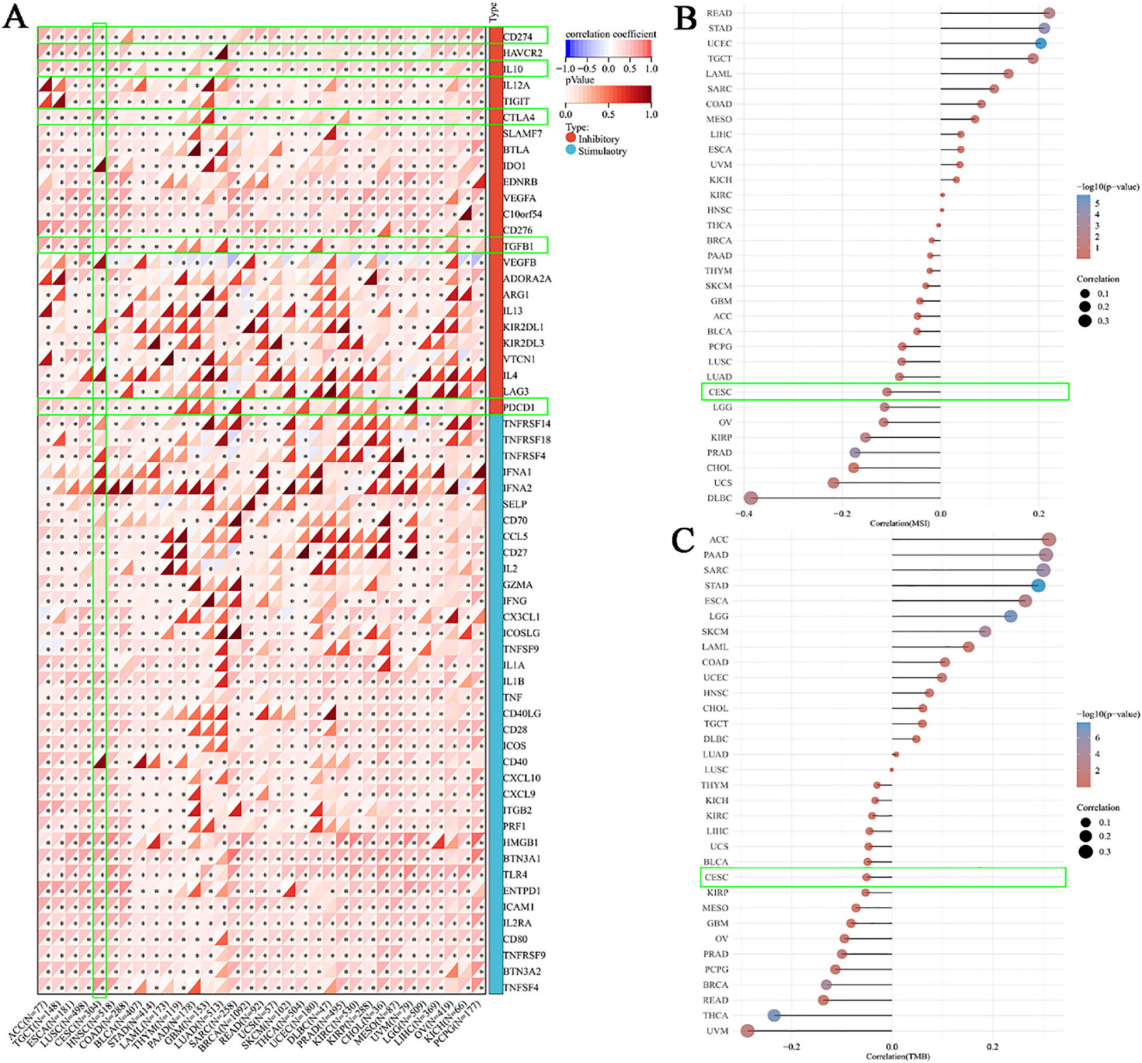
Correlation between BCL10 expression and Immune Checkpoint, MSI and TMB. **(A)** Correlation between BCL10 and immune-related genes (chemokines, receptors, MHC, immunoinhibitory, and immunostimulator). The x-axis represents different immune checkpoint genes, and the y-axis represents different tumor tissues. Each box in the plot represents the correlation analysis between the expression of the selected gene and the expression of immune checkpoint-related genes in the corresponding tumor. **(B)** Spearman correlation analysis of MSI and BCL10 expression. **(C)** Spearman correlation analysis of TMB and BCL10 expression. The horizontal axis in the figure represents the correlation coefficient between the gene and TMB, while the vertical axis represents different types of tumors. The size of the dots in the figure indicates the magnitude of the correlation coefficient, and different colors represent the significance of the p-value. In the illustrative diagram, the bluer the color, the smaller the *p*-value. * *p* < 0.05 with asterisks indicating the level of significance.

To further investigate the role of BCL10 in the TIME, we first explored the relationship between BCL10 and five categories of immune pathways: chemokines (41), chemokine receptors (18), MHC (21), immunoinhibitors (24), and immunostimulators (46). The results showed that BCL10 was positively correlated with most immune checkpoints in most tumors (**Figure 3A**). Notably, BCL10 was significantly positively correlated with the negative regulatory costimulatory molecules PD-1, CTLA4 and IL-10 on T cells in the CESC immune microenvironment. These results suggest that overexpression of BCL10 may regulate the activation and effector function of T cells by upregulating these negative regulatory costimulatory molecules, resulting in tumor immune tolerance and immune escape. In addition, we investigated the relationship between BCL10 expression levels and MSI and TMB. The results showed that BCL10 expression levels were negatively correlated with both MSI and TMB in the CESC immune microenvironment (**Figures 3B, C**, **Supplementary Figure 2**). The negative correlation between BCL10 and TMB/MSI suggests that BCL10-high tumors may exhibit reduced immunogenicity due to fewer neoantigens, compounded by PD-1/CTLA4-mediated T cell suppression. This dual effect could underlie their potential resistance to immunotherapy. This result suggests that tumors with high BCL10 expression are less immunogenic and may respond poorly to immunotherapy. BCL10 may be a potential therapeutic target, and inhibition of its expression or activity may enhance tumor immunogenicity and improve the efficacy of immunotherapy.

### 3.3 Bioinformatics analysis of the correlation between BCL10 expression and CD8+ T Cells, NF-κB signaling, and PD-1 in CESC

**Figure 4:**
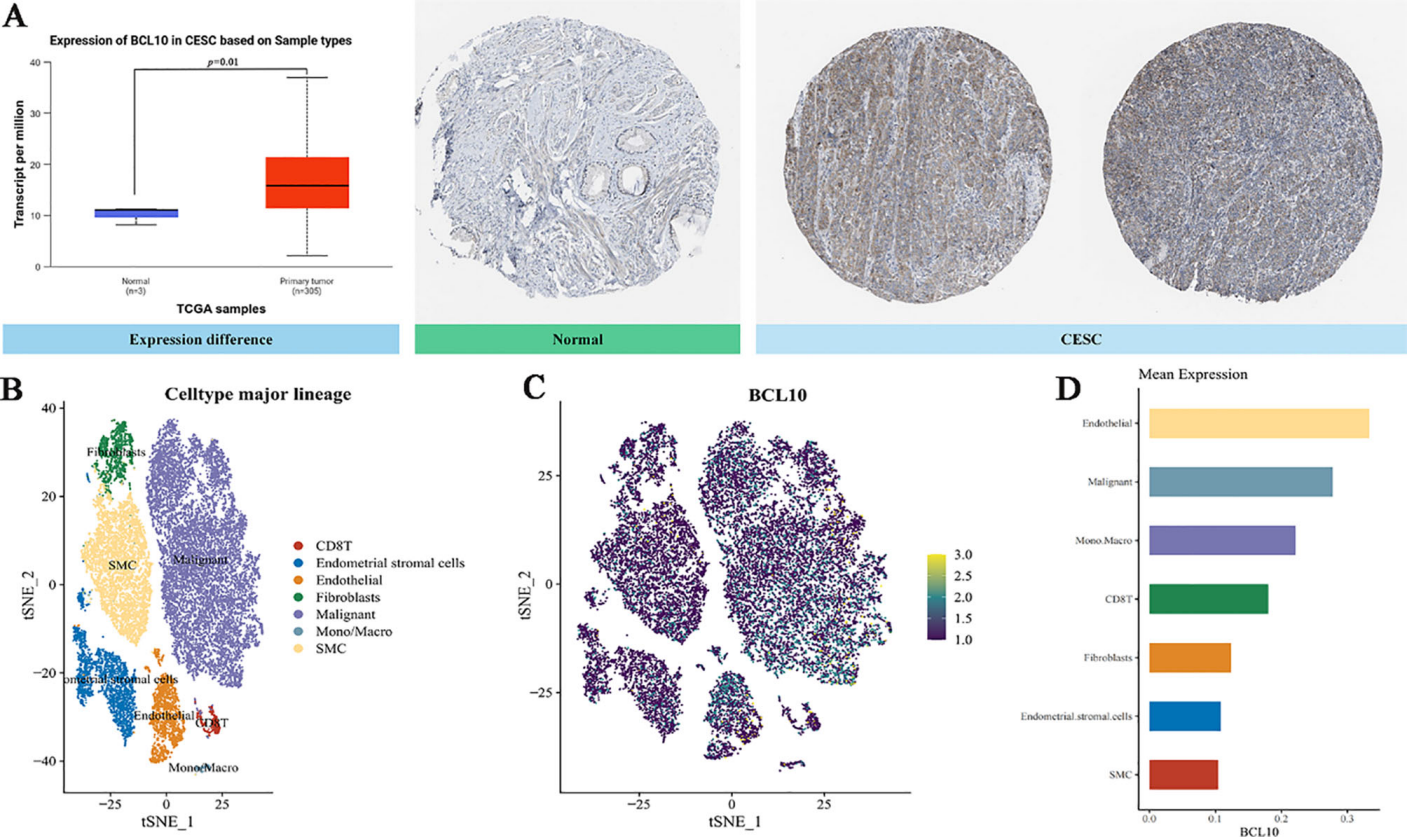
The expression of BCL10 in CESC and single cell sequencing analysis. **(A)** Comparison of BCL10 expression between normal(blue) and tumor tissues(red). BCL10 expression between normal and tumor tissues (left), immunohistochemistry images in normal (middle) and tumor tissues (right). **(B)** The t-SNE plot of single-cell clustering, where different colors represent different types of cells. **(C)** The t-SNE plot of the expression distribution of BCL10 in different cells, where different colors represent expression abundance. The darker the color, the lower the expression of the gene in the cell, and the brighter the color, the higher the expression of the gene in the cells. **(D)** The bar chart of the expression abundance of BCL10 in different cells.

In the aforementioned comprehensive analysis, we found that the expression of BCL10 was significantly negatively correlated with CD8+T cells in the CESC immune microenvironment. To further explore the role of BCL10 in the CESC immune microenvironment, we used the public database TCGA combined with immunohistochemical to confirm that BCL10 was significantly increased in CESC immune microenvironment(**Figure 4A**). Subsequently, we used the GEO database to analyze the single-cell RNA sequencing data of 20,938 cells in tumor tissue and adjacent normal tissue from a CESC patient (GSE168652). Cells clustering analysis based on t-distributed Stochastic Neighbor Embedding (t-SNE) algorithm, we identified seven main cell types through cell annotation: CD8+T cells, Endometrial stromal cells, Endothelial cells, Fibroblasts, Malignant cells, Monocyte (Mono) or Macrophage (Macro), Fibroblasts, and Smooth muscle cells (SMC) (**Figure 4B**). These results show that CD8+T cells are the most prominent immune infiltrating cells in the CESC microenvironment. Next, we compared the expression levels of BCL10 in the above seven different cell types. The results indicated that BCL10 expression was highest in CD8+ T cells and Mono/Macro cells among immune cells, and was primarily expressed in Endothelial and Malignant cells among non-immune cells(**Figures 4C, D**).

**Figure 5:**
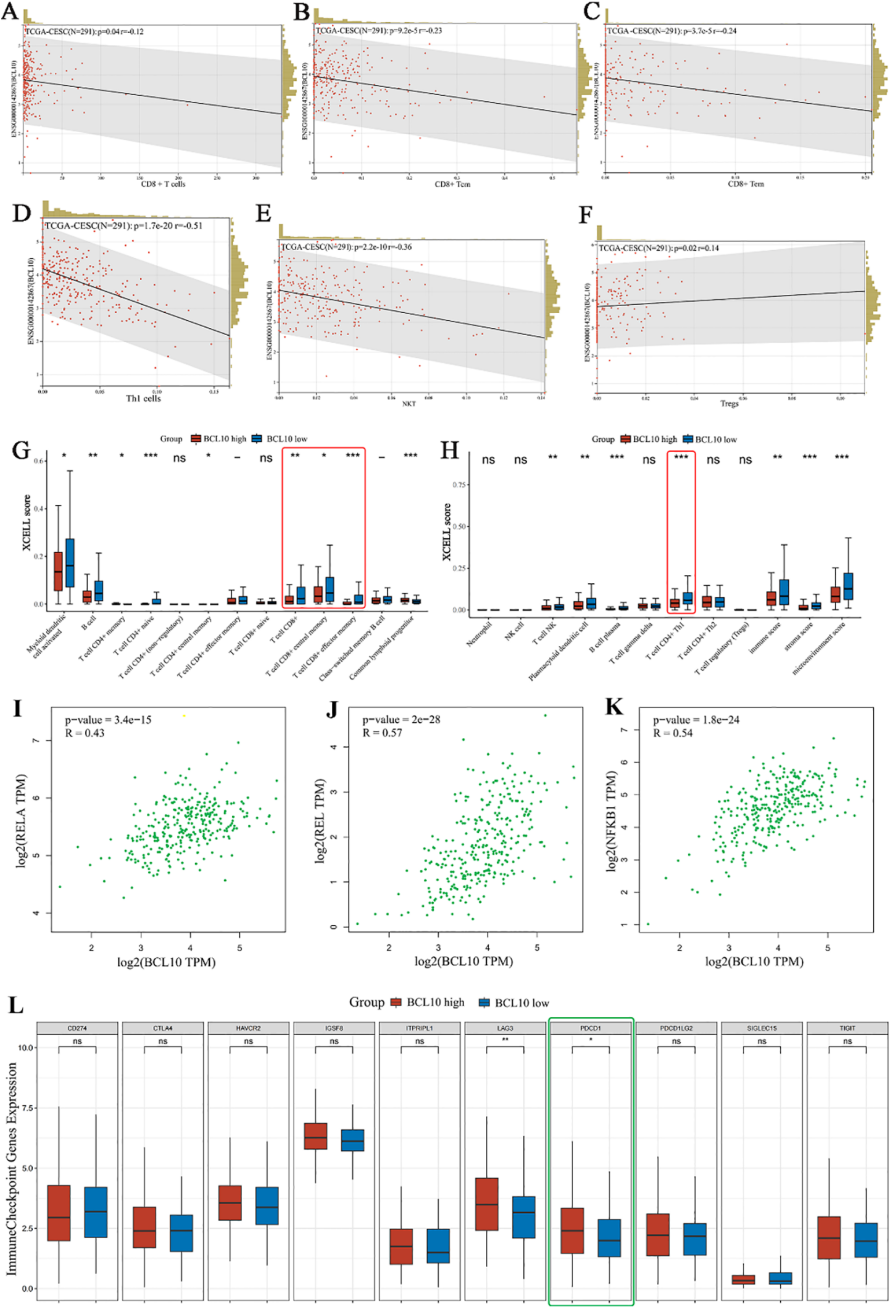
Correlation analysis of BCL10 expression with immune cell infiltration, NF-κB expression and PD1 expression in CESC tumor immune microenvironment. **(A–F)** Spearman correlation analysis chart, used to show the correlation between BCL10 expression and immune score. In the figure, each point represents a sample, the horizontal coordinate represents the immune score of the immune cell, and the vertical coordinate represents the expression level of the BCL10. The values at the top left represent Spearman correlation analysis results, including sample size, *p*-value, and correlation coefficient. **(G, H)** Distribution of immune scores of immune cells in the CESC immune microenvironment. The horizontal coordinate represents the type of immune cell infiltration, and the vertical coordinate represents the distribution of this type of immune infiltration score in different groups. Red represents the group with high BCL10 expression, and blue represents the group with low BCL10 expression. **(I–K)** Spearman correlation analysis plot, which is used to show the correlation between the expression of gene BCL10 with RELA,REL and NFKB1. In this plot, the x-axis represents the distribution of the expression of gene BCL10, and the y-axis represents the expression of RELA **(I)**、REL **(J)** and NFKB1 **(K)**. **(L)** The relationship between immune checkpoint expression and BCL10 high or low expression in CESC. The asterisk represents the level of significance, where *p < 0.05, **p < 0.01, ***p < 0.001. The significance of two sample groups is determined by the Wilcoxon test. ns, not statistically significant (p ≥ 0.05).

The results above indicate that CD8+ T cells are the most important immune infiltrating cells in the CESC immune microenvironment. Moreover, CD8+ T cells also exhibit significant BCL10 expression. To further analyze the correlation between BCL10 expression and the infiltration of various T cell subsets in the CESC immune microenvironment, we obtained relevant data from the TCGA and GTEx databases for analysis. The results further confirmed that the infiltration level of CD8+T cells in the CESC immune microenvironment was negatively correlated with BC10 expression, and high expression of BCL10 inhibited the infiltration of CD8+T cells in tumor microenvironment (*p*=0.04, R=-0.12) (**Figure 5A**). In addition, BCL10 expression was negative correlation between with CD8+ Central Memory T cells (*p*=9.2e-5, R=-0.23) (**Figure 5B**), CD8+ Effective Memory T cells (*p*=3.7e-5, R=-0.24) (**Figure 5C**), CD4+Th1 cells(*p* =1.7e-20, R=-0.51) (**Figure 5D**) and NK T cells (*p*=2.2e-10, R=-0.36) (**Figure 5E**). However, BCL10 expression was significantly positively correlated with Treg cells (*p* =0.02, R=0.14) (**Figure 5F**). We further analyzed the distribution of immune cell infiltration between BCL10 high expression (N=153) and low expression (N=153) groups in the CESC immune microenvironment. The results showed that the immune infiltration of CD8+T and CD4+Th1 cells was significantly inhibited in BCL10 high expression group (**Figures 5G-H**).

To further explore the potential mechanisms by which BCL10 regulates CD8+ T cell survival and effector function, we first obtained STAR-counts data and corresponding clinical information for CESC tumors from the TCGA database. We then employed single-sample gene set enrichment analysis (ssGSEA) and Spearman correlation analysis to investigate the correlation between BCL10 expression and pathway scores. The results showed that BCL10 was most closely related to the NF-κB signaling pathway. BCL10 expression was significantly positively correlated with RELA (*p*=3.4e-15, R=0.43), REL (*p* =2e-28, R=0.57) and NFKB1 (*p* =1.8e-24, R=0.54), the main signaling molecules of NF-κB pathway (**Figures 5I–K**). Meanwhile, a significant positive correlation was observed between BCL10 expression and the key upstream regulators of NF-κB signaling, CARD11 (*p*=0.00018, R=0.21) and MALT1 (*p*=8.3e-9, R=0.32) (**Supplementary Figures 3A, B**). Additionally, BCL10 expression showed a positive correlation with the P53 pathway, PI3K-AKT-mTOR pathway, TGFB, Glycolysis Gluconeogenesis, and Tumor proliferation signature in CESC (**Supplementary Figures 4A–E**). Conversely, it exhibited a significant negative correlation with Ubiquinone and other terpenoid quinone biosynthesis, as well as the Tumor Inflammation Signature (**Supplementary Figures 4F-G**).

As illustrated in **Figure 2**, within the immune microenvironment of most tumors, BCL10 exhibited positive correlations with five types of immune pathways: chemokines (n=41), chemokine receptors (n=18), MHC (n=21), immunoinhibitors (n=24), and immunostimulators (n=46). Notably, in CESC, BCL10 was significantly positively correlated with PD-1. To further investigate the role of PD-1 in BCL10-regulated T cell involvement in tumor immunity in CESC, we utilized the TCGA database to examine the relationship between BCL10 expression levels (high vs. low) and negatively regulated T cell surface costimulatory molecules, including CD274, CTLA4, HAVCR2, LAG3, PDCD1, PDCD1LG2, TIGIT, and SIGLEC15, within the CESC immune microenvironment. Our analysis revealed that the expression of T cell immune checkpoint genes LAG3 and PDCD1 was significantly higher in the BCL10 high expression group compared to the BCL10 low expression group (**Figure 5L**). This suggests that BCL10 may inhibit the infiltration of CD8+T cells, CD4+Th1 cells, and NKT cells in the TIME by increasing the expression of LAG3 and PDCD1, thereby promoting the progression of CESC.

### 3.4 Experimental verification of the association between BCL10 expression and CD8+ T Cells, NFKB, and PD1 in CESC

**Figure 6:**
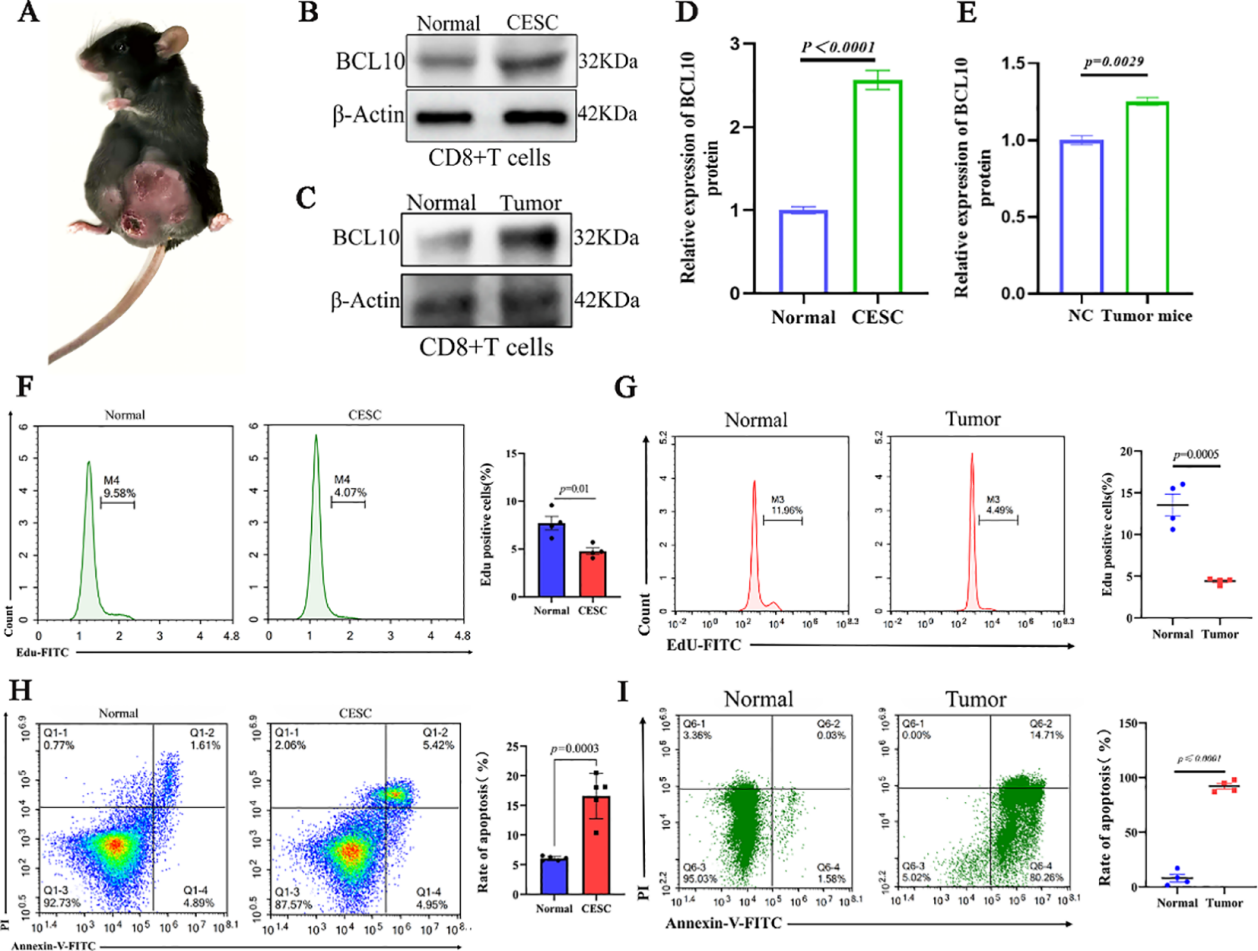
BCL10 inhibited proliferation and promoted apoptosis in CD8+T cell in CESC mouse model *in vivo*. **(A)** The mice model of CESC tumors. **(B)** The expression of BCL10 in CD8+T cells of normal and CESC mice. **(C)** Statistical chart of BCL10 after gray analysis. **(D)** The expression of BCL10 in CD8+T cells of normal and metastatic CESC mice. **(E)** Statistical chart of BCL10 after gray analysis. **(F)** Detecting the proliferation of CD8+T cells by flow cytometry with Edu staining in normal and CESC mice. **(G)** Detecting the proliferation of CD8+T cells by flow cytometry with Edu staining in normal and metastatic CESC mice. **(H)** Annexin V/PI double staining was used to detect the CD8+T cells apoptosis in normal and CESC mice. **(I)** Annexin V/PI double staining was used to detect the CD8+T cells apoptosis in normal and metastatic CESC mice. Summary data are mean ± standard deviation (SD), with *p* values being determined by two-tailed Student’s t-test.

To further elucidate the role of BCL10 in CD8+ T cells within the CESC immune microenvironment, we successfully established an implanted CESC tumor mouse model. This was accomplished by subcutaneously injecting TC-1 cells, which harbor the HPV16 E6/E7 genes, into the inguinal region of 6- to 8-week-old female C57BL/6J mice (**Figure 6A**). CD8+ T cells were isolated from the spleens of mice using magnetic beads. Subsequently, the expression levels of the BCL10 protein in CD8+ T cells were assessed for each experimental group. The results demonstrated that BCL10 expression in CD8+ T cells from CESC mice was significantly higher than that in cells from normal mice (**Figures 6B, D**). Subsequently, we detected the proliferation of CD8+T cells by flow cytometry with Edu staining, and the results showed that the proliferation of CD8+T cells in CESC model mice was significantly lower than that in normal mice (**Figure 6F**). Cell apoptosis was assessed using Annexin V/PI double staining. The results revealed that the proportion of apoptotic CD8+ T cells in CESC model mice was significantly higher than that in normal mice (**Figure 6H**). Moreover, we established a model of CESC abdominal metastasis for detection and obtained results similar to those observed in CESC. BCL10 was highly expressed in metastatic tumors (**Figures 6C, E**) and significantly inhibited the proliferation of CD8+T cells (**Figure 6G**). Additionally, high expression of BCL10 caused significant apoptosis and depletion of CD8+T cells (**Figure 6I**).

**Figure 7:**
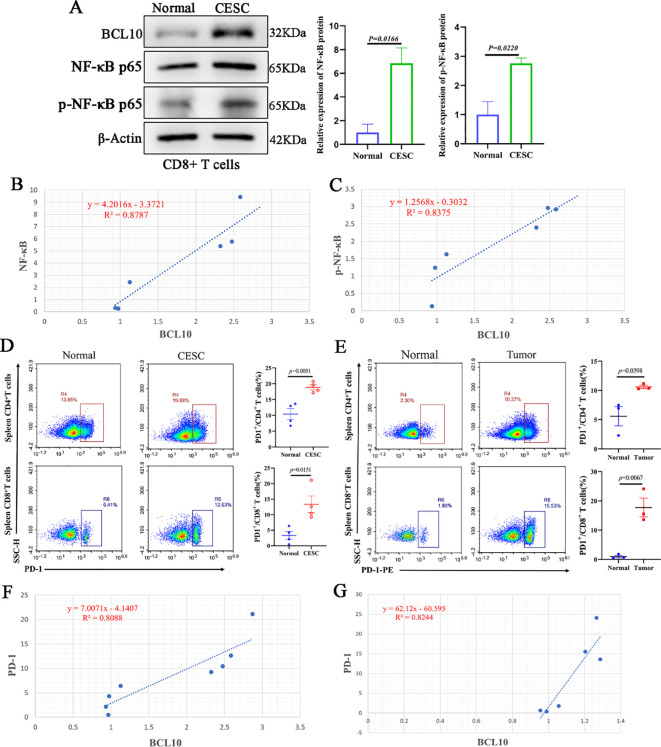
Overexpression of BCL10 is associated with activation of NF-κB and up-regulation of PD-1 expression in CESC. **(A)** The expression of BCL10, NF-κB p65 and p- NF-κB p65 in CD8+T cells of normal and tumor-bearing mice was detected by Western blot. **(B, C)** The correlation analysis of BCL10 and NF-κB and p- NF-κB expression in CD8+T cells. **(D)** The flow cytometry was used to detect the expression levels of PD-1 in CD4+T cells and CD8+T cells in CESC model mice and normal mice. **(E)** The flow cytometry was used to detect the expression levels of PD-1 in CD4+T cells and CD8+T cells in metastatic CESC mice and normal mice. **(F)** The relationship between BCL10 expression with PD-1 level in CESC. **(G)** The relationship between BCL10 expression with PD-1 level in metastatic CESC. Summary data are mean ± standard deviation (SD), with *p* values being determined by two-tailed Student’s t-test.

CD8+ T cells were extracted from each group of mice for Western blot analysis and correlation assessment. The results indicated that the protein levels of NF-κB and phosphorylated NF-κB (p-NF-κB) in CD8+ T cells from the CESC model mice were significantly elevated compared to those from normal mice (**Figure 7A**). Furthermore, correlation analysis revealed a positive association between BCL10 expression and the expression levels of NF-κB and p-NF-κB (**Figures 7B, C**).

Subsequently, flow cytometry was utilized to assess the expression levels of PD-1 in CD4+ T cells and CD8+ T cells isolated from CESC (cervical squamous cell carcinoma) model mice and normal mice. The results demonstrated that the proportion of PD-1 expression in both CD4+ T cells and CD8+ T cells was significantly higher in CESC model mice compared to normal mice (**Figure 7D**). Correlation analysis showed that the expression of BCL10 protein in CD8+T cells was positively correlated with PD-1 levels (**Figure 7E**). Similarly, the results from the CESC peritoneal metastasis mouse model indicated that the proportion of PD-1 in CD4+ T cells and CD8+T cells was significantly elevated (**Figure 7F**). Furthermore, the expression of BCL10 protein in CD8+T cells showed a positive correlation with the PD-1 level (**Figure 7G**).

## 4 Discussion

In this study, comprehensive bioinformatics and experimental analyses were performed to elucidate the role of BCL10 in shaping the tumor immune microenvironment (TIME). Multi-omics profiling revealed that BCL10 is significantly overexpressed in TIME across most cancers, strongly correlating with immune dysfunction. BCL10 upregulation suppresses the infiltration of effector immune cells, including CD8+ T cells, CD4+ Th1 cells, and NK T cells, while concurrently promoting the expansion of immunosuppressive populations such as Tregs and CD4+ Th2 cells. In CESC, single-cell RNA sequencing further demonstrated that high BCL10 expression directly impairs CD8+ T cell activity. Notably, in the implanted CESC mice model, elevated BCL10 levels in CD8+ T cells were associated with T cell exhaustion: proliferation arrest and apoptosis, PD-1 upregulation. The bioinformatics analysis and *in vivo* verification revealed an immunosuppressive axis orchestrated by BCL10 in tumor immune microenvironment. Through integrative deconvolution of bulk transcriptomic data via four independent algorithms (xCELL, CIBERSORT, QUANTISEQ, and MCP-counter), we identified a striking dichotomy in BCL10-associated immune cell infiltration within TIME. Specifically, BCL10 expression exhibited negative correlations with cytotoxic effector populations, including CD8+ T cells, CD4+ Th1 cells, and NK T cells, while demonstrating positive associations with immunosuppressive components such as Tregs and CD4+ Th2 cells (**Figure 2**). These computational findings collectively indicate that BCL10 may orchestrate an immunosuppressive TIME by dual mechanisms: suppressing anti-tumor immunity *via* cytotoxic cell inhibition and amplifying immune tolerance through Treg/CD4+ Th2 cells potentiation (46, 47). Evidence has established that BCL10 plays a pivotal role in maintaining the immunosuppressive function of regulatory T cells (Tregs) through its regulation of MALT1 paracaspase activity (47).

BCL10 plays a dual role in different stages of tumor immunity. BCL10 acts as a core component of the CBM complex (CARMA1-BCL10-MALT1), mediating NF-κB-dependent immune regulation through dynamic functional switching during tumor immunoediting (29, 48). During the initial immune surveillance phase, BCL10 controls NF-κB activation through phosphorylation of the CARMA1-IKK, thereby enhancing anti-tumor immunity (49, 50). At this phase, BCL10 facilitates antigen-driven T cell activation and promotes IL-2 secretion (49, 51). However, under chronic tumor antigen stimulation, sustained BCL10 upregulation paradoxically induces excessive activation of NF-κB, leading to T cell exhaustion (52, 53). ssGSEA and correlation analysis identified strong positive associations between BCL10 expression and key NF-κB components (RELA, REL, NFKB1; **Figures 5I–K**) (CARMA1 and MALT1; **Supplementary Figures 3A, B**). Elevated NF-κB and phosphorylated NF-κB protein levels was correlating strongly with BCL10 expression in CD8+ T cells isolated from CESC model mice (**Figure 7**). The single-cell sequencing analysis in CESC showed that despite CD8+T cells being the main immune infiltrates, BCL10 is highly expressed in CD8+T cells and inhibits the immune function of CD8+ T cells (**Figures 4**, **5**). Compared with normal mice, implanted CESC mice CD8+T cells significantly increased BCL10 and demonstrated 25% reduction in proliferating CD8+T cells and 3.2-fold increase in apoptotic subsets compared to controls (**Figure 6**). In CESC murine models with abdominal metastases, CD8+ T cells also significantly increased BCL10 and demonstrated 60% reduction in proliferating CD8+T cells and 47.5-fold increase in apoptotic subsets compared to controls (**Figure 6**). This quantitative immunosuppression of cytotoxic potential mechanistically explains the tumor’s immune-evasive phenotype. These findings extend prior reports of BCL10-mediated immunosuppression in malignant melanoma suggesting a cancer immunosuppressive axis (47). Sustained overexpression of BCL-10 may trigger exhaustion programs through chronic NF-κB stimulation, as observed in tumor-infiltrating lymphocytes from human prostate cancer (54), colitis-associated cancer (55)and melanoma tumor (56, 57). The PD-1 signaling pathway is a central regulator of tumor immune evasion, mediating T cell exhaustion and functional suppression (58, 59). In the experiments, elevated PD-1 expression was observed in CD8+ T cells isolated from CESC mouse models, strongly correlating with BCL10 levels (**Figure 7**). It suggests that BCL10 may drive tumor immune escape by upregulating PD-1 (5, 60, 61). These findings establish a mechanistic cascade: BCL10↑ → NF-κB/PD-1↑ → CD8+ T cell exhaustion, BCL10 hyperactivates NF-κB in CD8+ T cells, impairing their proliferation and effector functions (62). In CESC, sustained BCL10 overexpression in CD8+ T cells triggers NF-κB-mediated PD-1 induction, impairing cytotoxic function and survival, thereby shaping an immunosuppressive TIME. Such dysregulation weakens the anti-tumor immune response by inducing T cell exhaustion and fostering immune evasion (63, 64). Moreover, the negative correlation between BCL10 and TMB/MSI suggests that BCL10-high tumors may exhibit reduced immunogenicity due to fewer neoantigens, compounded by PD-1/CTLA4-mediated T cell suppression. This dual effect could underlie their potential resistance to immunotherapy. Building on our findings that BCL10 drives immune evasion through NF-κB-mediated PD-1 upregulation, several targeted strategies emerge. First, small-molecule inhibitors disrupting the CBM complex could abrogate BCL10 signaling, with precedent from MI-2 in lymphoma models (65). Second, shRNA nanoparticles targeting BCL10 mRNA may achieve selective knockdown while minimizing systemic effects, as demonstrated in pancreatic cancer (66). Importantly, our data suggest BCL10 inhibition could potentiate anti-PD-1 therapy by simultaneously reducing checkpoint ligand expression (**Figure 5L**) and reversing T cell exhaustion (**Figure 6**).

However, immune escape in tumors arises from the synergistic interplay of multiple mechanisms. In this study, we focused on the high expression of BCL10 induced by prolonged tumor antigen stimulation, which drives excessive NF-κB activation, resulting in elevated PD-1 levels and subsequent exhaustion of CD8+ T cells within the tumor immune microenvironment of cervical squamous cell carcinoma (CESC). While this mechanism represents a critical factor in tumor immunosuppression, it is neither isolated nor exclusive. Moving forward, we aim to investigate additional interacting mechanisms and explore the synergistic effects of multiple immune checkpoints. Specifically, we will examine whether BCL10 contributes to T cell exhaustion by modulating other immune checkpoints such as LAG-3, elucidate the impact of BCL10 overexpression on immune cells including regulatory T cells (Tregs) and natural killer T cells (NKTs), and analyze the roles of metabolic reprogramming (e.g., glycolysis inhibition and mitochondrial transfer) and immunosuppressive factors (e.g., TGF-β) in BCL10-mediated T cell exhaustion. While our study demonstrates BCL10’s association with CD8+ T cell dysfunction in preclinical models, future work should validate these findings in clinical cohorts by correlating BCL10 IHC scores with CD8+ T cell infiltration and patient survival data.

In conclusion, long-term stimulation with tumor antigens might lead to increased expression of BCL10, and suppress CD8+ T cells, Th1 cells, and NKT cells while promoting Treg and Th2 cell expansion across multiple tumor types. In CESC, high-expressed BCL10 is associated with hyperactivating NF-κB signaling and upregulating PD-1 expression in CD8+ T cell. These findings substantiate that upregulation of BCL10 exerts immunosuppressive effects by attenuating cell-mediated immunity within the TIME, particularly through impairing CD8+ T cell activity.

## Data Availability

The original contributions presented in the study are included in the article/**Supplementary Material**. Further inquiries can be directed to the corresponding authors.
