# Supplementary material for: High-expression of BCL10 inhibits cell-mediated immunity within the tumor immune microenvironment
Source: Front Immunol. 2025 Jun 5;16:1616321. doi: 10.3389/fimmu.2025.1616321 (PMC12176738; doi:10.3389/fimmu.2025.1616321)
Supplement: Supplementary file 1 [file Table1.docx]

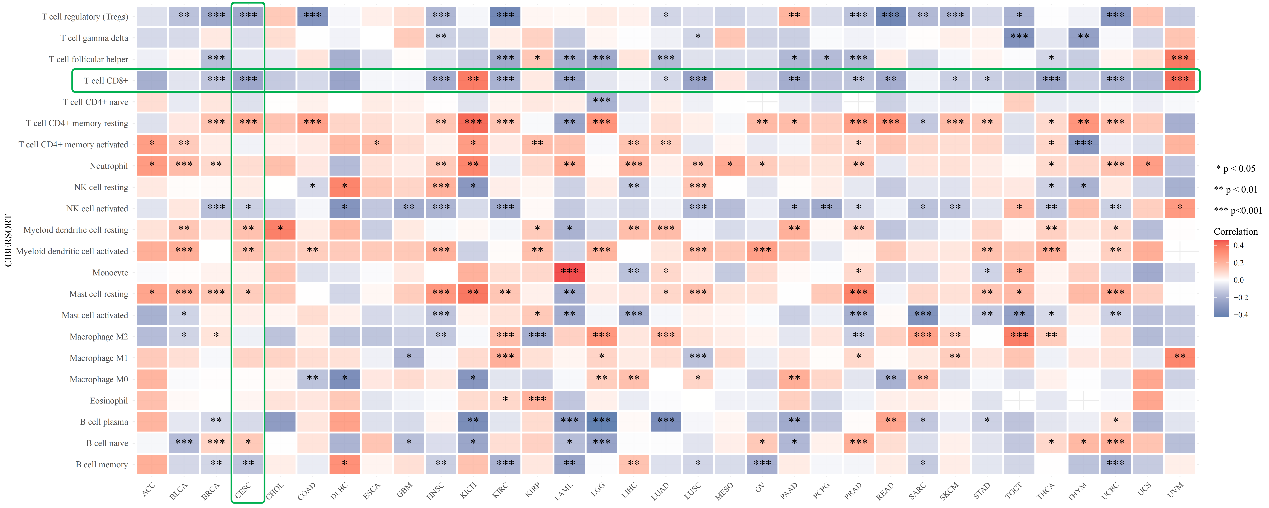


**Supplementary Figure 1:** Correlation between BCL10 expression and the level of immune cell infiltration in different types of cancer. Heatmap of pertinence between the expression of BCL10 and the level of immune infiltration in 33 types of cancer using CIBERSOR. The x-axis represents different tumor tissues, and the y-axis represents different immune infiltration scores. Different colors represent the correlation coefficients, with negative values indicating negative correlation and positive values indicating positive correlation. The stronger the correlation, the deeper the color. **p* < 0.05, ***p* < 0.01, ****p* < 0.001, with asterisks indicating the level of significance.


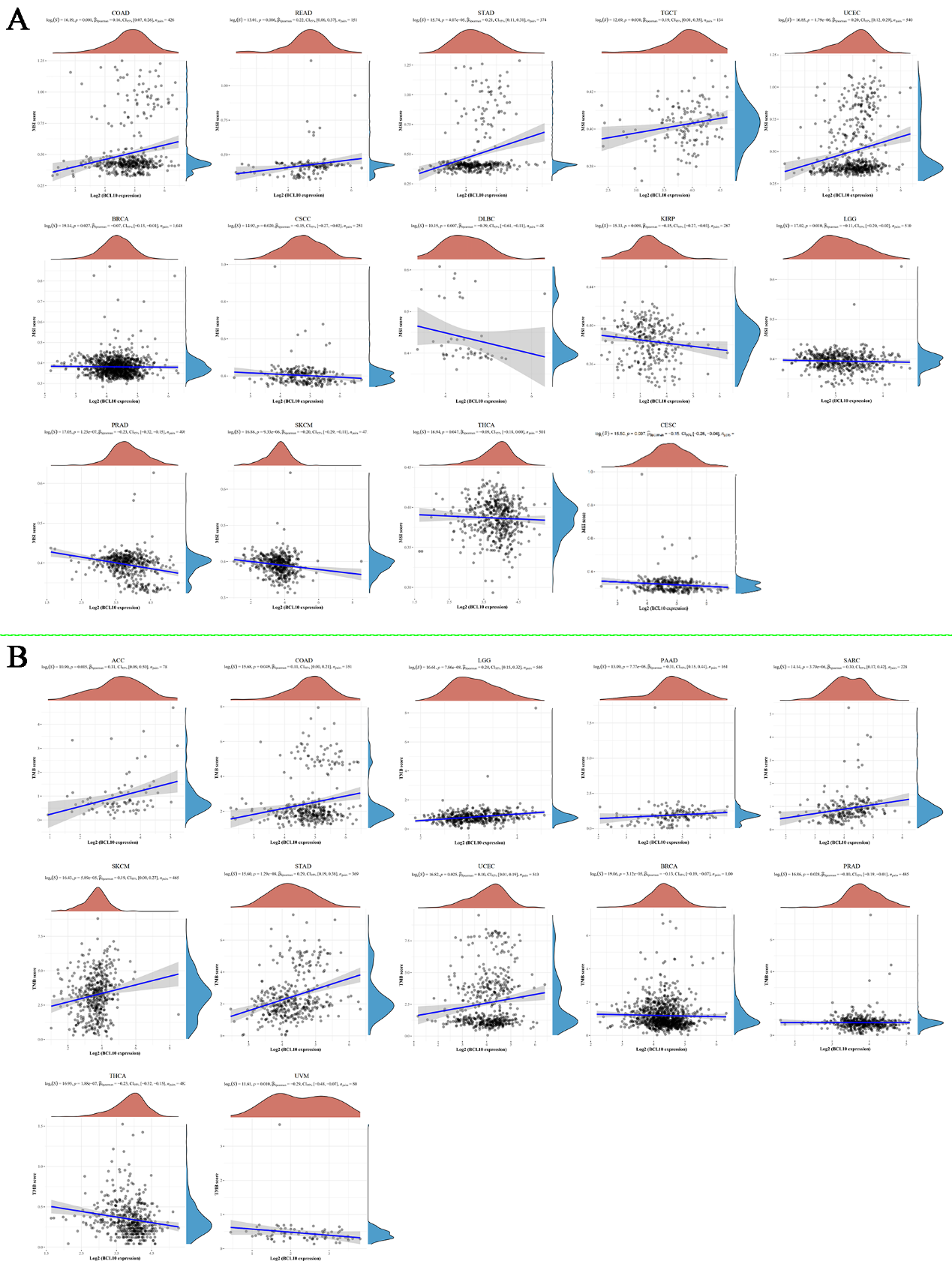


**Supplementary Figure 2:** Relationship between BCL10 expression and MSI and TMB in pan-cancer. **(A)** Relationship between BCL10 expression and MSI in 14 types of cancer. **(B)** Relationship between BCL10 expression and TMB in 13 types of cancer. In the figure, the x-axis represents the distribution of BCL10 expression, and the y-axis represents the score distribution of TMB/MSI. The density curve on the right depicts the distribution trend of TMB/MSI scores, while the density curve on the top shows the distribution trend of gene expression. The top of the figure displays the p-value, correlation coefficient, and the method used for correlation calculation.


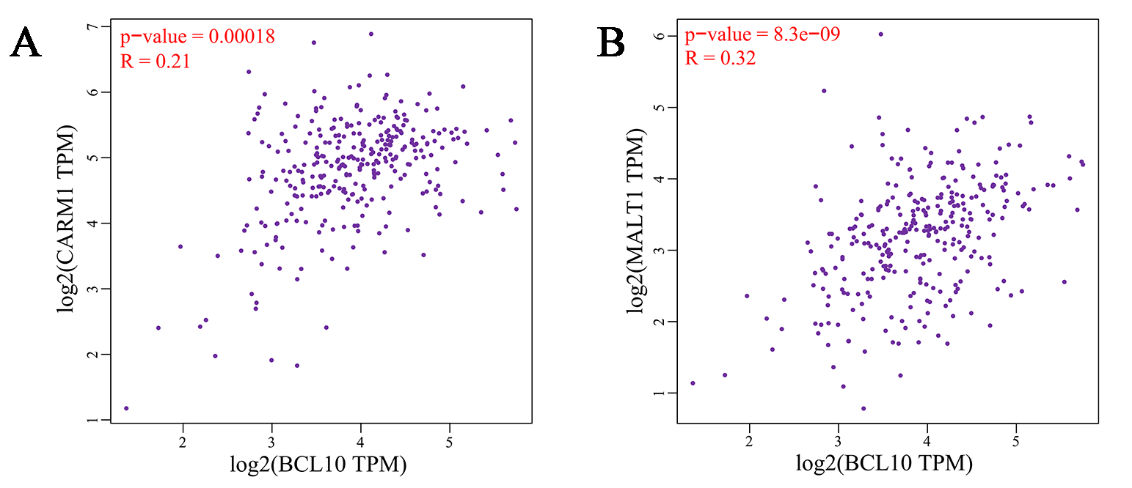


**Supplementary Figure 3:** Spearman correlation analysis of the relationship between BCL10 expression and key upstream regulators of NF-κB signaling. BCL10 expression showed significant positive correlations with CARM1 (A) and MALT1 (B).


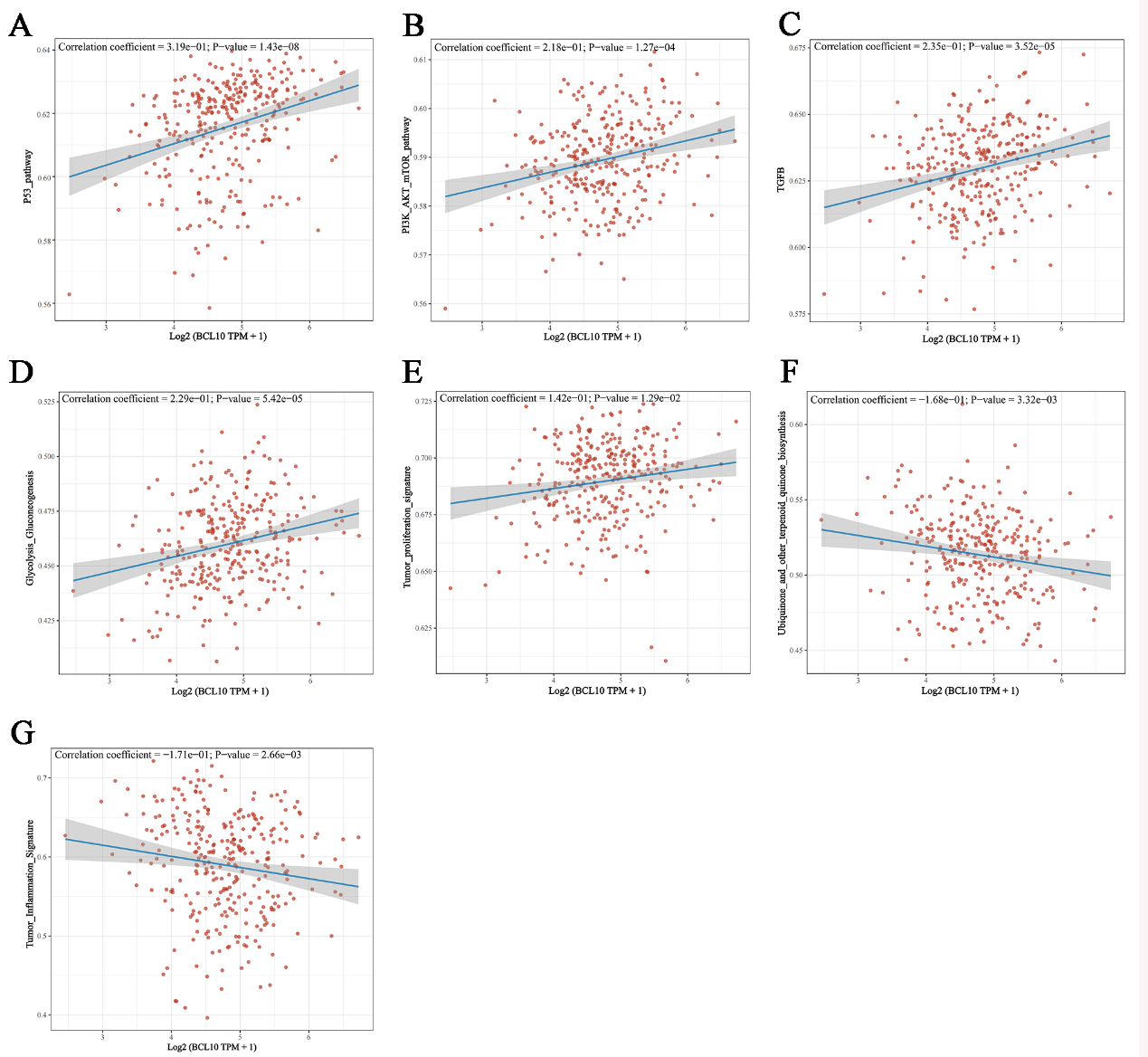


**Supplementary Figure 4:**  The Spearman correlation analysis plot, which is used to show the correlation between the pathway score and the expression of BCL10. In this plot, the x-axis represents the distribution of the expression of BCL10, and the y-axis represents the distribution of the pathway score. The density curve on the right shows the distribution trend of the pathway score, while the density curve on the top represents the distribution trend of the expression of BCL10. The values at the top represent the results of the Spearman correlation analysis, including the *p*-value, correlation coefficient, and correlation calculation method.
